# Long-Term Undesirable Consequences of Penile Skin Island Flap to Correct Penoescrotal Transposition: A Case Report and Review of Literature

**DOI:** 10.1155/2021/6656540

**Published:** 2021-01-11

**Authors:** Raquel Varea-Malo, Daniel Martínez Revuelta, Felix Campos-Juanatey, Paola Calleja Hermosa, Miguel Angel Correas Gómez

**Affiliations:** ^1^Urology Department, Marqués de Valdecilla University Hospital, Santander, Cantabria, Spain; ^2^GP Department, Marqués de Valdecilla University Hospital, Santander, Cantabria, Spain; ^3^Valdecilla Research Institute (IDIVAL), Santander, Cantabria, Spain; ^4^School of Medicine, University of Cantabria, Santander, Cantabria, Spain

## Abstract

Hypospadias is a congenital malformation of the male lower urinary tract, consisting of a ventral urethral opening proximal to the glans penis. This condition is corrected surgically in the paediatric age, with a great variety of techniques available. Traditionally, a tubularized genital skin was used for one- or two-stage repairs. Nowadays, the tendency is to use preputial or oral mucosa grafts, dorsally located, to avoid diverticula formation and prevent hair growth in the neourethra. We present a case of a patient born with proximal hypospadias with penoscrotal transposition, surgically corrected in his childhood, using dorsal penile skin island flap. The patient is referred to urology consultation in his adulthood for a weak urinary stream, recurrent infections, and a large amount of hair exiting through the urethral meatus.

## 1. Introduction

Hypospadias is the most common congenital malformation of the male lower urinary tract [1]. In this condition, the urethral opening is ventrally located, proximal to the tip of the penis. Its position ranges from a glandular position—located in the ventral aspect of the glans—to more proximal locations—proximal penile, penoscrotal, or even perineal. In severe cases, it is associated with penoscrotal transposition, where the scrotal insertion is above the penile base.

The hypospadias is corrected surgically during the early years of life, with different techniques depending on meatal location. There are more than 300 procedures described for the surgical treatment of hypospadias. Traditionally, preputial or genital skin was used as a tube for performing repairs in one surgical time. Nowadays, the tendency shifted towards using preputial or oral mucosa grafts, dorsally located, in single or staged repairs [2]. In most cases, there is a lack of adequate long-term follow-up of patients. All this variety reflects the absence of consensus among paediatric surgeons and urologists about which techniques are the most suitable ones.

Our objective is to describe the long-term undesirable consequences of the surgical repair by dorsal penile island flap [3] for proximal hypospadias with penoscrotal transposition. Additionally, we will review the literature and discuss the main complications potentially developed by hypospadias patients in adolescence and adulthood and the variety of current treatments. We followed the CARE guidelines for case report communication [4].

## 2. Clinical Case

A 38-year-old male was referred to our clinic for lower urinary tract symptoms (LUTS) and hair growth protruding through the external urethral meatus. The patient was diagnosed as a newborn of proximal penile hypospadias with penoscrotal transposition. His condition was corrected by when he was 3 years old by ventral transposition of dorsal penile skin flap in a single surgical time, according to the technique described by Perovic and Vukadinovic [3].

He complained of recurrent episodes of urinary tract infections (UTI) for several months, weak urinary stream, and difficulty in sexual intercourse.

**Figure 1:**
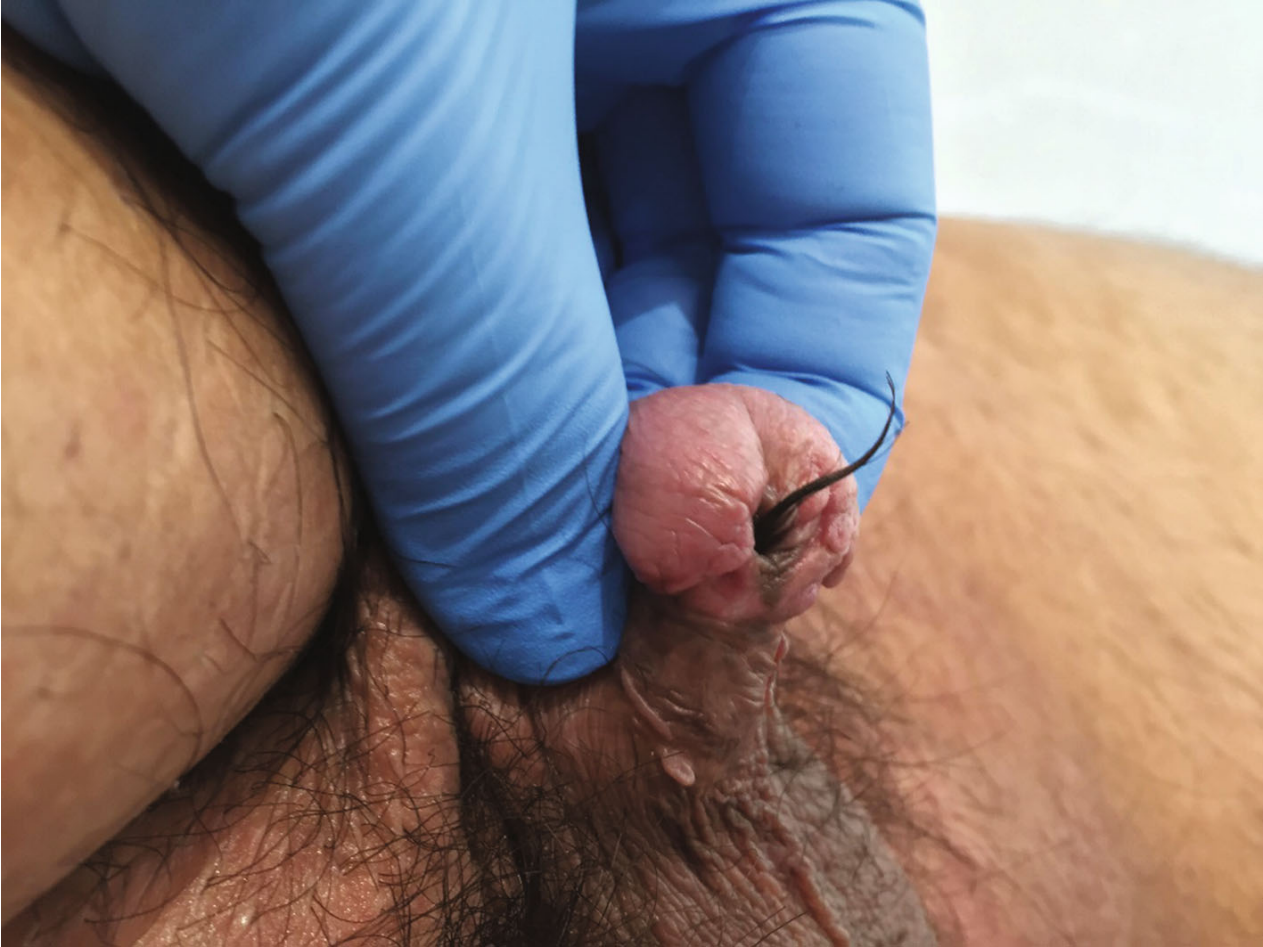
External appearance after dorsal penile island flap presenting a large amount of hair exiting the external meatus.

**Figure 2:**
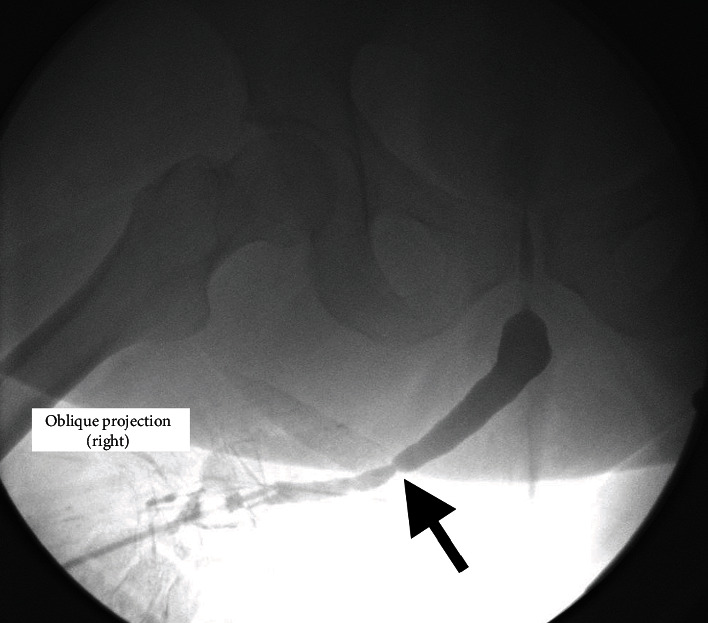
Retrograde urethrogram showing a stricture in the junction between the neourethra and the native bulbar urethra (arrow).

Physical examination confirmed the growth of large amounts of hair through the urethral opening (Figure 1). Urine culture was performed, yielding a negative result. Uroflowmetry showed a maximum flow rate (Qmax) of 11.1 ml/s, and an average flow rate of 7.9 ml/s for a voiding volume of 309 ml. The voiding curve evidenced a plateau shape, corresponding with urethral stricture. A retrograde urethrography (RUG) was performed, confirming the existence of a very short segment of urethral stricture, located at the junction of the penile and bulbar urethra (Figure 2). Flexible urethroscopy using a 16F cystoscopy was conducted, allowing for good passage through the penile neourethra to the penoscrotal angle, where the visualization of the stricture was not possible due to a large amount of hair.

After completing the diagnostic workout and offering a surgical approach for reconstruction, the patient rejected further interventions. He is being monitored, with voiding parameters maintained (Qmax above 10 ml/s) and no UTIs. His main complaints are related to sexual intercourse, due to penile curvature 45° downwards, and cosmetic alteration caused by severe scarring associated with the presence of hair through the urethra.

## 3. Discussion

Hypospadias is a congenital malformation of the male lower urinary tract, consisting of a urethral meatus opening proximally to the normal location at the distal end of the glans. Its incidence is approximately 1 in 300 men (0.3-0.45%) [1], with a family-specific genetic predisposition. It presents a great variability in terms of its severity, and the meatus can be located at various levels from the distal part of the penile shaft to the perineal area. It is frequently associated with ventral penile curvature, both at rest and in erection. There are several classifications for hypospadias. Classically, they are divided into proximal and distal. The proximal hypospadias (PH) are the most complex and can be perineal, interscrotal, penoscrotal, or midshaft. The distal hypospadias (DH), which are more frequent, can be subcoronal, coronal, or glandular. Additionally, the GMS classification (Glans, Meatus and Shaft) lists from 1 to 4 each item: G (glans size and urethral plate quality), M (meatal location), and S (penile curvature). Applying this classification before surgery is recommended since it has been correlated with the appearance of surgical complications [5].

The treatment of hypospadias is surgical, performing surgery preferably before the age of 5 [6]. The purpose of the interventions is threefold: bring the neomeatus to the tip of the glans to achieve correct urination and ejaculation, achieve cosmetic appearance suitable and satisfactory for the patient, and correct penile curvature in erection [7]. There is no ideal surgical technique, with more than 300 options described, making it very difficult to establish a standardized guide for treatment and follow-up.

Repair of hypospadias in childhood can lead to certain postoperative complications involving urinary urethra, bulbar or penile stricture, fistula, diverticulum, growth of hair, cavernous bodies, ventral chordee, axial torsion, glans, dehiscence or partial necrosis, and morphology of the penis and scrotal skin [8]. In most patients, short-term complications of primary repair are caused by surgical errors of design, technique, or postoperative care such as infection, suture dehiscence, urine extravasation, hematoma, ischemia, or postoperative necrosis in transplanted tissue [9]. However, they can also provide complications many years later, with successful functional and cosmetic results in primary repair and urethral stricture development decades after initial surgery [9]. There are not many studies on the long-term outcomes of hypospadias surgery, with patients lost to follow-up usually being higher than 50% [10]. In addition, only a few cases maintained an established protocol of prolonged routine checks over time. This is important since pubertal growth can change the cosmetic and functional aspects of the penis, with the appearance of new curvatures, affecting psychosexual development [11]. This is especially relevant in repairs using hair-bearing skin, like scrotal tubes or dorsal penile skin as in our case [3]. We have not found any specific study in the literature about the growth of large amounts of hair through the urethra, following a surgical repair of hypospadias with penoscrotal transposition by island skin flap of the dorsum of the penis.

Classical evaluation of the outcomes after hypospadias surgery is performed by comparing the early development of fistulas, diverticula, and strictures. However, there is usually no long-term follow-up for these patients [6, 12]. If the urinary function is resolved, there is usually no follow-up in terms of cosmetic result or sexual function. Often during the transition to adulthood, these patients are not referred to a urologist, and on the other hand, not all urologists are prepared to attend to the failed hypospadias, which aggravates the problem. It is estimated that 1 in 15 patients followed over 3 years will need reintervention [12].

With only one surgical intervention, about 75% of good results are obtained [12]. In the case of PH, these results are even worse, with complications reaching 58% (including 55.7% fistulas) in a series of 115 patients [10]. In other series, complications are less common, reporting 3.5-10% restrictures and 11-20% fistulas in patients with DH. In HP, an average of 3.7 surgeries (from 1 to 15) are required until a full resolution is achieved. Reinterventions occur during the first year in nearly 50% of the cases, but up to 37.7% are needed more than 2 years after the initial surgery [12].

In the follow-up of patients after hypospadias surgery, voiding pattern should be assessed to rule out obstructive symptoms. It is recommended to complete an evaluation with uroflowmetry, an objective, reliable, noninvasive, and inexpensive test. Flow rates could be altered in up to 1 in 3 patients, being a suitable test for screening of recurrences or other anatomic alterations, such as diverticula or hairballs [10].

Up to 60% of patients present long-term problems, being more prevalent in PH and in those operated using multistage techniques [7]. “Spraying,” or dispersion of the urine stream when leaving the urethral neomeatus, is usually the most reported symptom [6]. Another common symptom, reported by up to 50% of severe hypospadias, is postmicturition dribbling, due to the characteristics of the neourethra, which lacks adequate musculature for its complete emptying after voiding [7]. RUG usually associated with voiding cystourethrography or flexible urethroscopy is used to diagnose urethral complications, as we evidenced in our case. Targeted questionnaires are also used to specifically assess voiding quality. Ideally, they should be performed pre- and postsurgery to compare the results. From the voiding point of view, we can use the IPSS (International Prostate Symptoms Score) and urethral stricture PROM (Patient Reported Outcome Measure) questionnaires [10].

The cosmetic outcome should also be evaluated after hypospadias correction [13]. Several strategies and tools have been proposed. The HOSE (Hypospadias Objective Scoring Evaluation) [14] questionnaire, GPS-J (Junior Genital Perception Scale) [15], and PPS (Penile Perception Score) [16] are the most widespread questionnaires. PPS was first described for the paediatric population, being the first validated instrument for evaluation of the aesthetic result after hypospadias surgery [17]. A good correlation was found between evaluations by patients, parents, and urologists [16]. Another useful tool for comparing and evaluating the results is standardized photography, which has also been applied for developing rating scales [18].

Sexual problems in these patients are often present. They can be functional due to genital scarring or residual or relapsed penile curvature causing painful intercourse. Also, the body image disorder related to previous genital surgeries could lead to erectile dysfunction or ejaculation problems [7]. In addition, if the neomeatus has remained proximal to the balanopreputial sulcus, alterations in fertility may occur by not reaching the ejaculation in the cervix. This could be associated with weak ejaculation due to a lack of contractility of the neourethra.

The most common aspects to correct in adulthood are strictures, fistulas, diverticula, and persistent hypospadic location of the neomeatus [8]. Several aspects made the reconstruction complex [11]: poorly vascularized tissues after previous surgeries, congenital absence of corpus spongiosum, lack of skin and coverage tissues, and psychological issues of patients, avoiding further interventions, as we presented in our case.

Strictures are usually located at the junction of the native hypospadic urethra with the reconstructed area or in the distal neourethra. We should discuss whether urethral dilations are suitable for maintaining long-term patency or a new surgical intervention is required. In some cases, increasing urethral calibre by dorsal or ventral augmentation using grafts or flaps is feasible [11]. However, it is usually required to perform a complete urethral reconstruction after removing previous repairs. This complete substitution could be done in selected cases with oral mucosa grafts in a single procedure [19], but in most situations would require a staged approach using oral mucosa, preputial, or extragenital skin [11]. As a general rule, it is safer to remove all the fibrotic tissues, put wide grafts of oral mucosa—or other tissue—to cover the defect, and bring the new urethral plaque to the tip of the glans in a first procedure. After a period of 3 to 6 months, this graft can be tubularized to shape the neourethra, but the placement of more grafts prior to tubularization is required in approximately 20% of cases. For this reason, the term “multistage surgery” is preferred over the classic term of two-stage surgery. As the most common sexual problem is penile curvature, this aspect should be evaluated during reconstructive procedures, especially in PH. If necessary, dorsal albuginea application techniques could be used in paediatric population to correct curvatures [10]. In adulthood, releasing of the scarred tissues and ventral corpora cavernosum incisions at the time of reconstruction are preferred, to preserve penile length.

## 4. Conclusion

Long-term complications of hypospadias repair are common, but a large amount of hair growing in the neourethra is a rare consequence of penoscrotal transposition repairs. During a diagnostic workout, other alterations, as strictures, fistulas, or diverticula, should be considered. Long-term follow-up after hypospadias surgery should be offered, ideally until puberty or adulthood.
